# A New Species of Mesonivirus from the Northern Territory, Australia

**DOI:** 10.1371/journal.pone.0091103

**Published:** 2014-03-26

**Authors:** David Warrilow, Daniel Watterson, Roy A. Hall, Steven S. Davis, Richard Weir, Nina Kurucz, Peter Whelan, Richard Allcock, Sonja Hall-Mendelin, Caitlin A. O’Brien, Jody Hobson-Peters

**Affiliations:** 1 Public Health Virology Laboratory, Forensic and Scientific Services, Department of Health, Archerfield, Queensland, Australia; 2 Australian Infectious Diseases Research Centre, School of Chemistry and Molecular Biosciences, The University of Queensland, St Lucia, Queensland, Australia; 3 Berrimah Veterinary Labs, Department of Primary Industries and Fisheries, Darwin, Northern Territory, Australia; 4 Centre for Disease Control, Health Protection Division, Northern Territory Department of Health, Darwin, Northern Territory, Australia; 5 LotteryWest State Biomedical Facility, Genomics, School of Pathology and Laboratory Medicine, University of Western Australia, Perth, Western Australia; 6 Department of Clinical Immunology, Pathwest Laboratory Medicine Western Australia, Royal Perth Hospital, Perth, Western Australia, Australia; University of California Davis, United States of America

## Abstract

Here we describe Casuarina virus (CASV), a new virus in the family *Mesoniviridae*. This is the first report of a mesonivirus in Australia, which extends the geographical range of this virus family to 3 continents. The virus was isolated in 2010 from *Coquillettidia xanthogaster* mosquitoes during surveillance in the suburbs of Darwin, the capital of the Northern Territory. Cryo-electron microscopy of the CASV virions revealed spherical particles of 65 nm in size with large club-shaped projections of approximately 15 nm in length. The new virus was most closely related to *Alphamesonivirus 1*, the only currently recognized species in the family. In 2013 a further 5 putative new mesonivirus species were described: Hana, Méno, Nsé, Moumo and Dak Nong viruses. The evolutionary distance between CASV and two of its closest relatives, Cavally and Hana viruses (Jones-Taylor-Thornton distance of 0.151 and 0.224, respectively), along with its isolation from a different genus of mosquitoes captured on a separate continent indicate that CASV is a new species.

## Introduction

The viral taxonomic order *Nidovirales* is a genetically diverse group of enveloped single-stranded positive-sense RNA viruses consisting of the families *Arteriviridae*, *Roniviridae*, *Coronaviridae*, and the recently established *Mesoniviridae*. The latter currently has one member species which was first described in 2009 [1] and later characterized in more detail in isolates from mosquitoes trapped on two continents. Cavally virus (CavV) was obtained from the Cote d’Ivoire region of West Africa from several mosquito species predominantly from the genus *Culex* [2], whilst Nam Dinh virus (NDiV) was isolated from *Culex vishnui* and *Culex tritaeniorhynchus* mosquitoes from northern and central Vietnam [3]. These viruses are the first members of the order known to infect insects [2], [3]. Both viruses were sufficiently similar to allow their classification as a single species, *Alphamesonivirus 1* [4]. Membership of the family is likely set to expand due to the recent isolation of a number of viruses sufficiently different to justify the inclusion of additional species [5], [6].

The genome organization for the mesoniviruses is ORF1a-ORF1b-ORF2a-ORF2b-ORF3a-ORF3b, with all mesoniviruses except Méno virus (MenoV) coding for an additional ORF-4 [2]–[4], [6]. Translation of ORF1a generates polyprotein 1a (pp1a) and expression of ORF1b, which encodes the RNA-dependent RNA polymerase and other replicase-related domains, is regulated by a putative ribosomal frameshift (RFS) motif with sequence GGAUUUU [2], [3], which results in read-through of the ribosome translating ORF1a and production of the polyprotein 1ab (pp1ab). Individual functional proteins are processed from the polyproteins by the viral protease 3CL^pro^ and a putative host-cell protease(s) [6]. Translation of the ORFs further downstream is from subgenomic mRNA transcripts. Mesoniviruses replicate in A*edes albopictus* (C6/36) insect cells and induce varying degrees of cytopathic effect (CPE) depending on the virus [2], [6], [7]. There is a large variation in the reported particle size of 60–80 [3], 80 [5], [7] or 120 nm [2], [6] and in the appearance of the surface projections. While the projections of CavV and others isolated from Cote d’Ivoire have large club-shaped projections of approximately 12 nm, electron microscopy analysis of NDiV from culture supernatant revealed short spikes of only 3–4 nm [7].

The mesoniviruses have no known disease association but have attracted interest primarily as they represent an evolutionary bridge across a large gap in RNA genome size [3], [8]. They are descended from the ancestral group that acquired a 3′-to-5′ exoribonuclease activity (ExoN) which improved replication fidelity sufficiently to enable the evolutionary leap to the largest known RNA genome size of the coronaviruses. Genome structure for this order of viruses can roughly be divided into ORF1a, ORF1b and the 3′ORFs which each encode for separate functional domains leading to suggestions of a genomic division of labor [8]. The expansion in genome size was not uniform and occurred in waves with increasing genome size: first ORF1b, then ORF1a and, finally, the 3′ORF region [8]. As the number of new member species of mesoniviruses increases, further light should be shed on the biochemical and genetic processes which enabled this expansion.

We report on the latest mesonivirus to be isolated from mosquitoes and have tentatively named this virus Casuarina virus (CASV) after the location of its isolation. CASV was isolated from *Coquillettidia xanthogaster* mosquitoes trapped at Casuarina in the Northern Territory, Australia in 2010. Cryo electron tomography of purified virions revealed that the particles were approximately 65 nm in diameter, with a smooth lipid bilayer and the presence of spike projections with a globular head attached to the virion by a low density stalk. Furthermore, treatment of the virions with a non-ionic detergent showed that the virus did not possess an internal core. Partial sequencing of the replication domains in ORF1a and ORF1b revealed a close relationship with alphamesonivirus 1, but the virus is sufficiently different to justify its classification as a new species of mesonivirus.

## Materials and Methods

### Ethics Statement

No specific permits were required for the described field studies and no specific permissions were required for the locations/activities for mosquito trapping because they are public lands and are not privately owned or protected. The sites of mosquito trapping are those where the Northern Territory Department of Health conducts regular mosquito monitoring and has done so for many years. These field studies did not involve endangered or protected species.

### Mosquito Trapping

Mosquitoes were trapped in June, 2010 in the Darwin suburb of Casuarina in the Northern Territory of Australia (Latitude 12°22.4′S, Longitude 130°52.9′E; approximately 14 km from Darwin city centre). The mosquitoes were trapped by Encephalitis Virus Surveillance (E.V.S.) dry ice baited light traps [9], sorted to species in pools of 1 to 50 as previously described [10] and stored at −80°C until processed. All of the mosquitoes were female. Mosquito pools were homogenized for RNA extraction and virus isolation as previously described [11].

### Cell Culture and Virus Isolation

*Aedes albopictus* C6/36 cells (ATCC CRL-1660) were cultured in MEM with 10% fetal bovine serum (FBS) and incubated at 25°C, which is the temperature at which the C6/36 cells are maintained at the particular laboratory within which the primary inoculations were performed. Initial virus isolation was performed from mosquito homogenate that was inoculated onto monolayers of the C6/36 cells and incubated at 25°C for 7 days. Culture supernatant (200 μL) was collected (passage 0) and aliquots of this culture supernatant were then stored at −80°C for further analysis.

Successive passaging of the virus was performed by inoculating onto monolayers of C6/36 cells grown in RPMI 1640 with 5% FBS and incubation at 28°C for 3–4 days before harvesting. The viral titre was determined by 50% tissue culture infective dose (TCID_50_) assays using the method of Reed and Muench (1938) [12]. Virus stocks were titrated by serial 10-fold dilution and inoculated onto monolayers of vero cells (for West Nile virus) or C6/36 cells (for CASV) as described by May *et al.* (2006) [13]. After five to six days incubation, wells exhibiting CPE were identified by microscopic analysis.

### Electron Microscopy

A passage 2 stock of CASV was inoculated onto C6/36 cells and the culture supernatant harvested 4 days post-infection by centrifugation at 2,000×g, for 15 min at 4°C. PEG 8,000 was added to the clarified supernatant to a final concentration of 5% and incubated overnight with gentle mixing at 4°C. The virus/PEG solution was pelleted at 12,000 rpm (JA16.250 rotor Beckman), for 1 h at 4°C, resuspended in cold NTE (12 mM Tris at pH 8, 120 mM NaCl, 1 mM EDTA pH8), prior to ultracentrifugation through a 20% sucrose cushion at 28,000 rpm (SW32Ti rotor Beckman) for 2 h at 4°C. Following overnight incubation of the pellet in NTE at 4°C, the resuspended pellet was layered onto a 10–40% potassium tartrate/glycerol gradient and centrifuged at 50,000 rpm (SW60Ti rotor, Beckman) for 1 h at 4°C. The virus band was extracted and buffer exchanged into NTE pH 8 prior to negative staining with 1% uranyl acetate and then viewed using a Tecnai T12 120 keV TEM operating at 120 kV.

### Collection of 2D Cryo TEM Data

Samples (4 μl) of gradient-purified virus were applied to glow-discharged holey carbon films supported on 400-mesh copper grids. Cryo-freezing was performed on a vitrobot (FEI) operating at 4°C and 100% humidity with a 6 second blot time. Two dimensional images were collected from vitrified specimens at liquid nitrogen temperature under low-dose conditions with a Tecnai T12 120 keV TEM operating at 120 kV, equipped with a 4 k FEI Eagle CCD Camera. Images were taken at 67000× magnification and binned by 2 for a final pixel size of 3.4Å. 2D Image analysis was performed using the IMOD software package.

### Cryo-electron Tomography

Tilt series were collected using a Tecnai F30 FEG-TEM (FEI) operating at 300 kV with a 4 k lens-coupled camera (Direct Electron). Tilt series data were collected over a range of ±60° at 1.5° increments along two orthogonal axes and recombined computationally to produce a 3D tomogram using the etomo software package.

### NP-40 Treatment of Virions

Gradient-purified CASV virions were treated with the non-ionic detergent NP-40 (Sigma) at dilutions ranging from 0.05%–1%. Treatments were assessed by electron microscopy after 6, 30 or 50 min incubation at 4°C by negative staining with uranyl acetate as described above.

### Structural Protein Analysis

Gradient-purified CASV virions were resuspended in the glycoprotein denaturing and reaction buffers (NEB) as per the manufacturer’s instructions, with or without the addition of 500 units PNGase F. Each preparation was incubated at 37°C for 1 h, prior to the addition of LDS sample buffer (Life Technologies) and separation on a 4–12% Bis-Tris SDS-PAGE gel (Life Technologies). The gel was stained using Sypro Ruby stain (Life Technologies) according to the manufacturer’s protocol and the protein bands were visualized on the Typhoon phosphoimager (GE Healthcare).

### Vertebrate and Insect Cell Infection Assays

The vertebrate cells, African Green Monkey Kidney (Vero; ATCC CCL-81), baby hamster kidney (BHK; ATCC CCL-10), human adeno carcinoma (SW-13 ATCC CCL-105) and chicken embryo fibroblast (DF-1; ATCC CRL-12203) were cultured in Dulbecco’s modified Eagle’s medium (DMEM) containing 2–10% FBS, while the Chao Ball (*Culex tarsalis*; obtained from Dr Robert Tesh, University of Texas Medical Branch (UTMB)) and HSU (*Culex quinquefasciatus* obtained from Dr Robert Tesh, UTMB) [14] cells were grown in Leibovitz’s L-15 medium supplemented with 10% tryptose phosphate broth and 15% FBS. All vertebrate cells were incubated at 37°C with 5% CO_2,_ while the insect cells were maintained at 28°C. All media were supplemented with 50 U penicillin/mL, 50 μg streptomycin/mL and 2 mM L-glutamine.

Monolayers of each cell line were inoculated with either CASV or with West Nile virus (WNV; Kunjin strain MRM61C or New York 99 strain 4132) at a multiplicity of infection (M.O.I) of 10. After incubation for 1 h, the inoculum was removed and the monolayer washed three times with sterile phosphate buffered saline (PBS). Cell-specific growth medium was added to each well and the cultures incubated for five days. Two additional blind passages were performed by inoculating freshly seeded cell monolayers with a 1/10 dilution of the culture at five day intervals. The monolayers were observed for morphological changes daily and the culture supernatant harvested at each passage was stored immediately at −80°C. The cell monolayers were harvested directly into tri reagent (Sigma) for subsequent RNA extraction, as per the manufacturer’s instructions, whereas RNA extraction from the supernatants was achieved with the Nucleospin RNA virus kit (Machery-Nagel).

Extracted RNA from the passage 1 cell monolayers were tested for CASV and control WNV viral RNA by reverse transcription PCR (RT-PCR) using the NidoF1(5′-GTTGTATGCTATGCCGYCG-3′)/CASVR1(5′- TATGTAGTGGGCTGTCATCG -3′) or FU2/cFD3 [15] primer sets, respectively, and the Superscript III One-step RT-PCR system with Platinum Taq DNA polymerase (Life Technologies). Cycling conditions for the NidoF1/CASVR1 primer set were 45°C/30 min; 94°C/2 min; followed by 40 cycles of 94°C/30 sec, 49°C/30 sec, 68°C/30 sec and final 68°C extension for 5 min. Cycling conditions for the FU2/cFD3 primer pair were identical apart for the following conditions for the 40 cycles of PCR amplification: 94°C/30 sec, 45°C/30 sec, 68°C/1 min. The PCR products were sequenced to confirm their identity.

Assessment of RNA extracted from P3 supernatant of CASV-inoculated cells was performed using the Rotor-Gene SYBR green RT-PCR kit (Qiagen), the primer pair CASVF1 (5′-CACACACCATCACCACAGCACACC-3′)/CASVR3 (5′-TATGCAAAAGCAAGCCGAATTCTGC-3′) and 5 μL RNA. The cycling conditions were 55°C/10 min; 95°C/5 min; followed by 40 cycles of 95°C/5 sec, 60°C/10 sec. The limit of detection of the assay was determined to be 10^2^ TCID_50_ equivalents/mL using RNA extracted from 10-fold serial dilutions of a titred stock of CASV as per the TCID_50_ assay described above.

The infectious titres of CASV and WNV in passage 1 C6/36 and Chao Ball cell culture supernatants were determined by TCID_50_ assays as described above. However, for consistency, WNV was also titred on C6/36 cells and the infectious titre determined by fixing the cell monolayer and performing an ELISA using a WNV-specific monoclonal antibody, as described in Clark et al., (2007) [16].

For IFA analysis, monolayers of C6/36 and Vero cells grown on glass coverslips were inoculated with CASV, WNV or mock-infected as described above. After 48 hrs, the cells were fixed in acetone and probed with a cocktail of two monoclonal antibodies that are specific for double-stranded RNA (dsRNA; O’Brien, Hobson-Peters, Hall et al., unpublished) using methods described previously [17].

### Sequence-independent Amplification

Amplification of viral nucleic acids was a modification of a previously described method [18]. Firstly, virus was purified from tissue culture supernatant by performing a clarifying centrifugation step (2,000×g; 10 min; room temperature) followed by filtration through a 0.22 μm filter. Free nucleic acids were digested by enzymatic treatment using RQ1 DNase (1 U/ml) and RNase ONE (2 U/ml) at 37°C for 30 min. Virus particles were sedimented using ultra-centrifugation (100,000 *g*; SwTi55 rotor; 2 h; 4°C) and resuspended in sterile PBS overnight on ice. Nucleic acids were extracted using a Qiaamp Viral RNA extraction kit (Qiagen) as per the manufacturer’s recommendations with the modification that the carrier RNA was omitted from the AVL buffer. The RNA (5 μl of extract) was used as template in a reverse transcription using a primer containing a random hexamer attached to an arbitrary sequence (C-6N 5′-GAGAAACCCACCACCAGANNNNNN-3′). This was followed by second strand DNA synthesis using the SuperScript III One-Step RT-PCR System with Platinum Taq High Fidelity mix (Invitrogen) with cycling [25°C for 5 min; 37°C for 30 min; 95°C for 2 min]_1_[95°C, 30 sec; 25°C, 30 sec; 72°C, 2 min]_5_. The product of this reaction (2 μl) was a template for amplification in a mix with a primer containing only the arbitrary sequence (Primer C 5′-GAGAAACCCACCACCAGA-3′) and AmpliTaq Gold thermostable DNA polymerase with cycling: [95°C, 12 min]_1_[95°C, 30 sec; 59°C, 30 sec; 72°C, 2 min]_50_[72°C, 7 min]_1_[15°C]_hold_.

### Library Preparation

DNA samples were sheared to approximately 230–250 bp fragments using an S2 sonicator (Covaris, Inc. MA, USA). Sequencing libraries were prepared with an IonXpress Plus Fragment Library Kit (Life Technologies, NY, USA). Individual samples were prepared using different adaptors containing a 10 bp “barcode” at the 3′ end, followed by a linker sequence (GAT) to allow electronic/bioinformatic discrimination following sequencing. Fragments of approximately 330 bp were excised from an agarose gel to ensure a high proportion of full-length sequencing templates and the libraries were quantified using a High Sensitivity DNA chip on a Bioanalyzer 2100 (Agilent Technologies, CA, USA).

### Template Preparation and Sequencing

Barcoded samples were pooled in equimolar ratios to a total concentration of 9 pM in low TE buffer. Template preparation and enrichment was performed using an Ion OneTouch Template 200 Kit (Life Technologies, NY, USA) on a OneTouch 2 and OneTouch ES (Life Technologies, NY, USA). Sequencing was performed using an Ion PGM 200 Sequencing Kit on “316” sequencing chips for a total of 500 nucleotide flows, yielding average read lengths of 220–230 bp. Five or six samples were pooled on a single chip, generally yielding >450,000 reads per sample.

### Sequence Assembly and Bioinformatics

A virus consensus sequence was assembled from the data using GeneiousPro v5.6 software and CavV (NCBI accession number NC_015668.1) as a reference sequence, trimming at least 30 nt from the 5′ end of each read, with the sensitivity set at the highest level. To construct phylogenetic trees, global multiple protein sequence alignment was performed with the Geneious Alignment feature (Blosum62 cost matrix; gap open penalty, 12; gap extension penalty, 3). MEGA 5.03 was used for phylogenetic analysis. The multiple sequence alignment was imported for estimation of the gamma parameter. The evolutionary distance was calculated from a concatenated domain alignment using the estimated gamma parameter (Jones-Taylor-Thornton model and complete gap deletion). An unrooted phylogenetic tree was then generated by the maximum likelihood method using the estimated gamma parameter (Jones-Taylor-Thornton model and complete gap deletion) with 1000 bootstrap replicates.

## Results and Discussion

### Isolation and Culture of CASV

During surveillance for viruses in June 2010, mosquitoes were trapped at various locations within the Northern Territory of Australia [17]. Of 94 pools of mosquitoes, which consisted of mosquitoes from five different genera [17], only one homogenate of pooled *Coquillettidia xanthogaster* female mosquitoes (n = 43) caused severe destruction of the cell monolayer when inoculated onto C6/36 cells. Sequencing of a 1200 bp PCR product obtained using random RT-PCR on extracted RNA, revealed that the virus was a mesonivirus (closest match using 843 bp input was Dak Nong virus (E value 9e-156); 77% identity with 89% query coverage) after comparing the sequence with the Genbank database using the BLASTn algorithm (http://www.ncbi.nlm.nih.gov/genbank). This virus was tentatively named Casuarina virus (CASV) after the suburb of Darwin (capital of the Northern Territory) within which the mosquitoes were trapped. This was the first isolation of a mesonivirus from *Coquillettidia spp.* mosquitoes and its identification in Australian mosquito populations reveals widespread distribution of the *Mesoniviridae* family.

### Virus Particle Morphology

**Figure 1:**
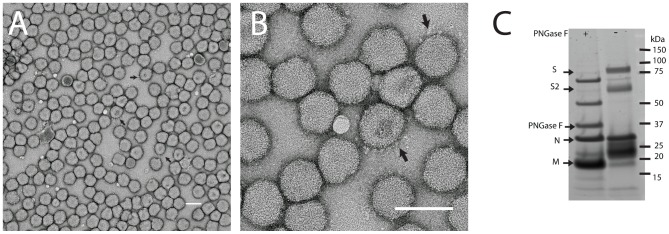
CASV morphology and structural proteins. (A,B) Negative-staining electron micrograph of CASV particles following potassium tartrate gradient purification and staining with 1% uranyl acetate. Visible spike protrusions are marked with arrows. Each scale bar represents 100 nm. (C) SDS-PAGE analysis of gradient-purified CASV virions. The virion proteins were assessed for the presence of N-linked glycans by treatment with PNGAse F (+) or left untreated (−). Putative protein identity is shown on the left of the image.

**Figure 2:**
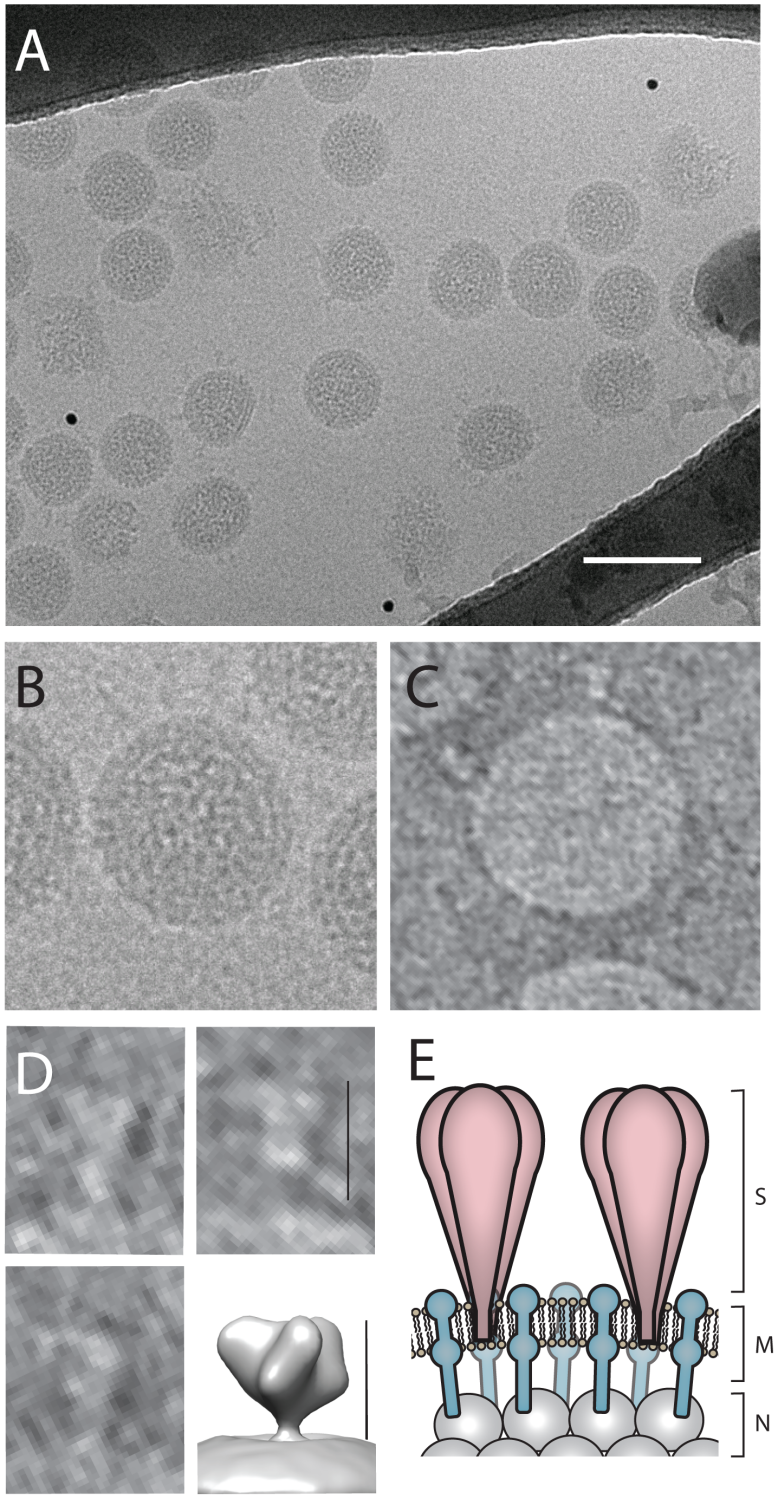
Cryo-electron imaging and in silico virion surface reconstruction. (A) Cryo-electron micrograph of purified CASV particles imbedded in vitrious ice layer within a holey carbon support. Scale bar in 100 nm (white). Representative virions shown for cryo-projection (B) and tomographic reconstruction (inverted contrast - C). Stratified surface densities at the virion surface due to M protein layer imbedded in virion envelope can be seen clearly together with an electron dense core containing the ribonucleoprotein (RNP) complex. (D) Representative surface projections extracted from a 10Å filtered tomogram show 15 nm spikes at the virion surface, comparable in dimensions to that observed for SARS coronavirus spike (shown in lower right inset – scale bars 15 nm. Refer to EMBD-1423 [28]). (E) Proposed virion architecture equivalent to that observed within the C*oronaviridae* family, with trimeric spike proteins projecting from an ordered M protein/lipid bilayer support. The RNP layer lies below a low density region but is tethered to the envelope through interactions with M protein projections.

Electron microscopy of negative-stained CASV virions revealed spherical particles of uniform size and appearance consistent with previous mesonivirus descriptions (Fig. 1)[2], [3], [5]–[7]. The particles had a mean diameter of 84 nm and some (but not all) particles carried large club-shaped surface projections similar to those described for CavV, Nsé virus (NséV), Hana virus (HanaV) and MénoV [2], [6] (Fig. 1, S1). To visualize the protrusions more clearly, cryo-electron microscopy was performed. Each virion displayed a smooth envelope containing a double density associated with the lipid bilayer which can be attributed to the membrane protein and an electron-dense core (Fig. 2). Images of 133 viruses were selected and the average diameter was determined to be 65 nm, in contrast to the 84 nm determined by uranyl acetate staining (Fig. S1). The difference in size determined by each of the protocols might be attributable to a flattening of the virus particles onto the grid during negative staining, thus giving the appearance of a larger virion diameter. Such differences in virion size have been observed by others previously when comparing images obtained following negative staining and freeze drying [19]. Again, protrusions were evident on some, but not all of the virions. In contrast to CavV and NséV, the protrusions of CASV did not appear as numerous or ordered, which may be unique to this virus, but might also be attributed to differences in staining or time of virus culture harvesting.

For a more detailed examination of the surface projections we utilized cryo-electron tomography to obtain 3D representations of the purified virions in a hydrated, vitreous ice environment. Tomographic analysis revealed spike-like projections on the surface of some of the virions with a characteristic globular head attached to the virion surface through a low density stalk (Fig. 2D; Movie S1). The spikes were approximately 15 nm in length which was comparable to the well-characterized severe acute respiratory syndrome coronavirus (SARS-CoV) and murine hepatitis virus (MHV) S proteins of 17 nm and 19 nm respectively [20]. The discrepancy of these observations compared to the previous estimations of 3–4 nm [7] for other mesonivirus spike length was most likely due to the use of alternative imaging methodologies and could be resolved by further investigations of other mesoniviruses using cryo-TEM which is more likely to preserve the native virion structure.

**Figure 3:**
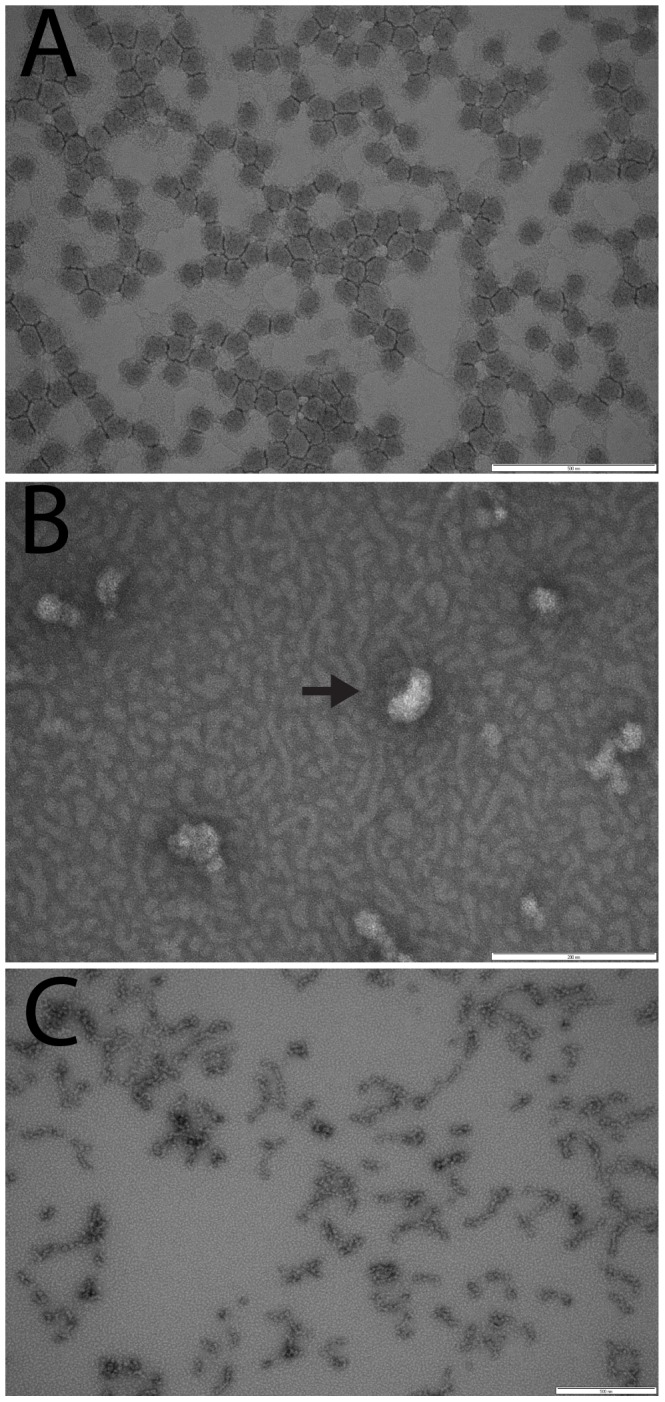
Analysis for mesonivirus core using NP-40 treatment. Gradient-purified CASV particles were left untreated (A) or treated with 0.05% NP-40 for 6 min (B) or 0.25% NP-40 for 30 min (C) to disrupt the lipid envelope. No proteainaceous internal component was released suggesting the absence of a core. An uncoating virion is highlighted in panel B with an arrow. Scale bars: A = 500 nm, B and C = 200 nm.

Some members of the order *Nidovirales*, such as transmissible gastroenteritis coronavirus, contain a core shell comprising membrane (M) and nucleocapsid (N) proteins that encases the viral RNA [21]. Whether mesoniviruses have a core has not been previously determined. To disrupt the viral envelope and enable visualization of the particle’s internal structure, purified CASV virions were treated with the non-ionic detergent NP-40. No proteinaceous structure was visible by electron microscopy following treatment (Fig. 3). Hence, CASV does not have an internal core.

Analysis of the CASV structural proteins by SDS PAGE gel electrophoresis showed four clearly defined proteins with apparent molecular weights of approximately 75, 57, 27 and 20 kDa (Fig. 1), consistent with the sizes of the mesonivirus glycoprotein spike proteins (S and S2), N and M proteins respectively [6]. A smear between 20–27 kDa, likely to be caused by the multiple glycosylated M products, probably obscured the visualization of the S1 protein band with a predicted size of 23 kDa. As expected, glycosylation of the S, S2 and M proteins was confirmed by the increased mobility of these proteins through the gel following removal of N-linked glycans using the glycosidase, PNGase F (Fig. 1).

### CASV does not Infect Vertebrate Cells

The absence of CavV isolation from male mosquitoes has prompted speculation that mesoniviruses may be transmitted by an alternative strategy to ovitransposition [2]. However, the inability of all mesoniviruses tested to date to replicate in vertebrate cells suggests that a vertebrate cryptic host is unlikely [5], [6]. Permissiveness for CASV to replicate in a range of insect and vertebrate cells was assessed by regularly monitoring inoculated cell monolayers for evidence of CPE and by assaying the cell monolayers and cell culture supernatant for the presence of viral RNA by RT-PCR. The ability of each of the cell lines to support the replication of other positive sense single stranded RNA viruses was also confirmed by the inoculation and subsequent RT-PCR detection of WNV RNA. While destruction of C6/36 cell monolayers by CASV was clearly evident by 2–3 days post-infection, there was no evidence of growth observed visually or by RT-PCR in any of the vertebrate cells tested, including an avian cell line (Table 1). In support of these data, IFA analysis of CASV-inoculated Vero and C6/36 cell monolayers with a cocktail of two monoclonal that are specific for dsRNA (O’Brien, Hobson-Peters, Hall et al., unpublished), showed that while there was distinct peri-nuclear staining in the CASV-inoculated C6/36 cells, consistent with the detection of dsRNA replication intermediates [22], there was no detectable replication of CASV in the Vero cells (Fig. S2A).

**Table 1:** Replication of viral isolates in various cell lines.

Cell line	Source	CASV			WNV	
		CPE	RT-PCR P1 lysate	Real time RT-PCR P3 supernatant	CPE	RT-PCR
**C6/36**	*Aedes albopictus*	**+**	**+**	**+**	**−**	**+**
**Chao Ball**	*Culex tarsalis*	**−**	**+**	**+**	**−**	**+**
**HSU**	*Culex quinquefasciatus*	**−**	**+**	**+**	**−**	**+**
**Vero**	Monkey	**−**	**−**	**−**	**+**	**+**
**BHK**	Hamster	**−**	**−**	**−**	**+**	**+**
**SW13**	Human	**−**	**−**	**−**	**+**	**+**
**DF-1**	Chicken	**−**	**−**	**−**	**+**	**+**

Although the replication of CASV in Chao Ball (*Culex tarsalis*) and HSU (*Culex quinquefasciatus*) cells was confirmed by RT-PCR, markedly less total PCR product was observed (Fig. S2B) and a lower infectious titre was confirmed for virus grown using Chao Ball cells compared with that from inoculated C6/36 cells (10^5.77^ TCID_50_ units/mL for Chao Ball vs 10^8.5^ TCID_50_ units/mL for C6/36 cells). A similar trend was also seen for WNV (10^5.65^ TCID_50_ units/mL for Chao Ball; 10^8.22^ TCID_50_ units/mL for C6/36). The surprising differences between the ability of CASV to replicate in two insect cell lines is consistent with observations by Kuwata et al., (2013) whereby significantly lower levels of replication of DKNV in the *Culex tritaeniorhynchus* cell line compared with that achieved in the C6/36 cell line were noted [5]. Differences in the growth of these mesoniviruses in *Culex* cells compared to C6/36 cells might be attributable to the defective innate immune response of the latter [23], [24]. C6/36 cells lack Dicer-2 activity – an important enzyme in the insect cell antiviral response [23]. Lack of this antiviral defense mechanism likely enhances the permissiveness of these cells to replication of some viruses. Inoculation of other *Aedes albopictus* cells such as RML12 or RNAi-competent U4.4 would be useful to clarify these observations [25]–[27].

### Analysis of CASV Predicted ORF Sequences

The complete genome of CASV, apart from the extreme 5′ and 3′ termini, was elucidated using sequence-independent amplification (Genbank accession number KJ125489). Analysis of the sequence revealed a putative RFS motif that was just upstream of the border between ORF1a and ORF1b (GGAUUUU). In these two ORFs, CASV had identical domain content and order as other recently identified mesoniviruses. This included the putative viral 3C-like chymotrypsin-like protease (3CL^pro^), RNA-dependent RNA polymerase (RdRp), helicase (HEL), exonuclease (ExoN), N7-methyl-transferase (NMT) and 2′-O-methyltransferase (OMT) domains. These domains also had a high level of amino acid sequence identity to homologous regions of the mesoniviruses (Table 2), particularly alphamesonivirus 1 with which it shared 93 and 94% identity in the RdRp and HEL domains, respectively. An amino acid identity of <90% has been suggested as a possible value for demarcation of species in the mesoniviruses based on a similar value within the coronaviruses [6]. The other 4 replicase domains showed less than 90% amino acid identity; hence, on the basis of sequence similarity alone it was unclear whether the new virus was a new species. However, as the majority of replicase identity scores were less than 90%, and as the evolutionary distance between CASV and the other mesoniviruses was considerably different, CASV is clearly a new species in the genus *Mesonivirus* in the family *Mesoniviridae*.

**Table 2:** Percentage amino acid identity of CASV replicase proteins with other mesoniviruses.

Domain	Region of ORF1ab^1^	CavV	NDiV	HanaV	NséV	MénoV	MoumoV	DKNV
**3CL^pro^**	1385–1688	84.9	85.5	81.6	76.6	70.7	ND	75.3
**RdRp**	3003–3458	94.1	94.1	88.4	82.1	75.9	84.2	90.4
**HEL**	3937–4280	93.6	92.7	87.2	82.8	86.0	82.6	92.4
**ExoN**	4357–4613	80.9	82.9	79.4	71.3	68.2	65.9	76.7
**NMT**	4614–4847	77.0	79.1	78.7	65.7	66.9	67.5	75.6
**OMT**	4848–5088	76.9	76.9	70.2	69.0	66.1	69.0	75.2

1 Numbering based on CavV amino acid sequence, NCBI accession number NC_015668.1. Other accession numbers: CASV, KJ125489; NDiV, DQ458789.2; HanaV, JQ957872.1; NséV, JQ957874.1; MénoV, JQ957873.1; MoumoV, KC768950.1; and DKNV, AB753015.1.

To further resolve the relationship of CASV to the other mesoniviruses, the evolutionary distances of the concatenated replicase domains 3CL^pro^, RdRp, HEL, ExoN and OMT were calculated for each of the mesoniviruses (Table 3). For the isolates CavV and NDiV, which constitute the species *Alphamesonivirus 1*, a value of 0.031 was obtained. This compares favourably with a value of 0.029 for the same regions calculated previously by Lauber et al. (2012) [4]. As expected, CASV was closest to alphamesonivirus 1, with distances of 0.147 and 0.151 separating it from NDiV and CavV, respectively. This was considerably larger than the minimum distance established for the demarcation of nidovirus species of 0.037 for this region [4]. Hence by this analysis, CASV is a new species in the family *Mesoniviridae*, and genus *Mesonivirus*. This analysis also confirmed that the newly identified HanaV, NséV, and Méno viruses are species of mesonivirus. From this analysis, Dak Nong virus (DKNV) should also be considered a new species in the genus. Unfortunately, insufficient sequence information was available for Moumo virus (MoumoV) to be able to determine its appropriate classification.

**Table 3:** The evolutionary distances separating the mesoniviruses.

	HanaV	NséV	MénoV	CavV	CASV	DKNV
**NDiV**	0.166	0.335	0.420	0.031	0.147	0.144
**HanaV**		0.376	0.497	0.164	0.224	0.223
**NséV**			0.439	0.336	0.330	0.396
**MénoV**				0.420	0.427	0.492
**CavV**					0.151	0.153
**CASV**						0.231

**Figure 4:**
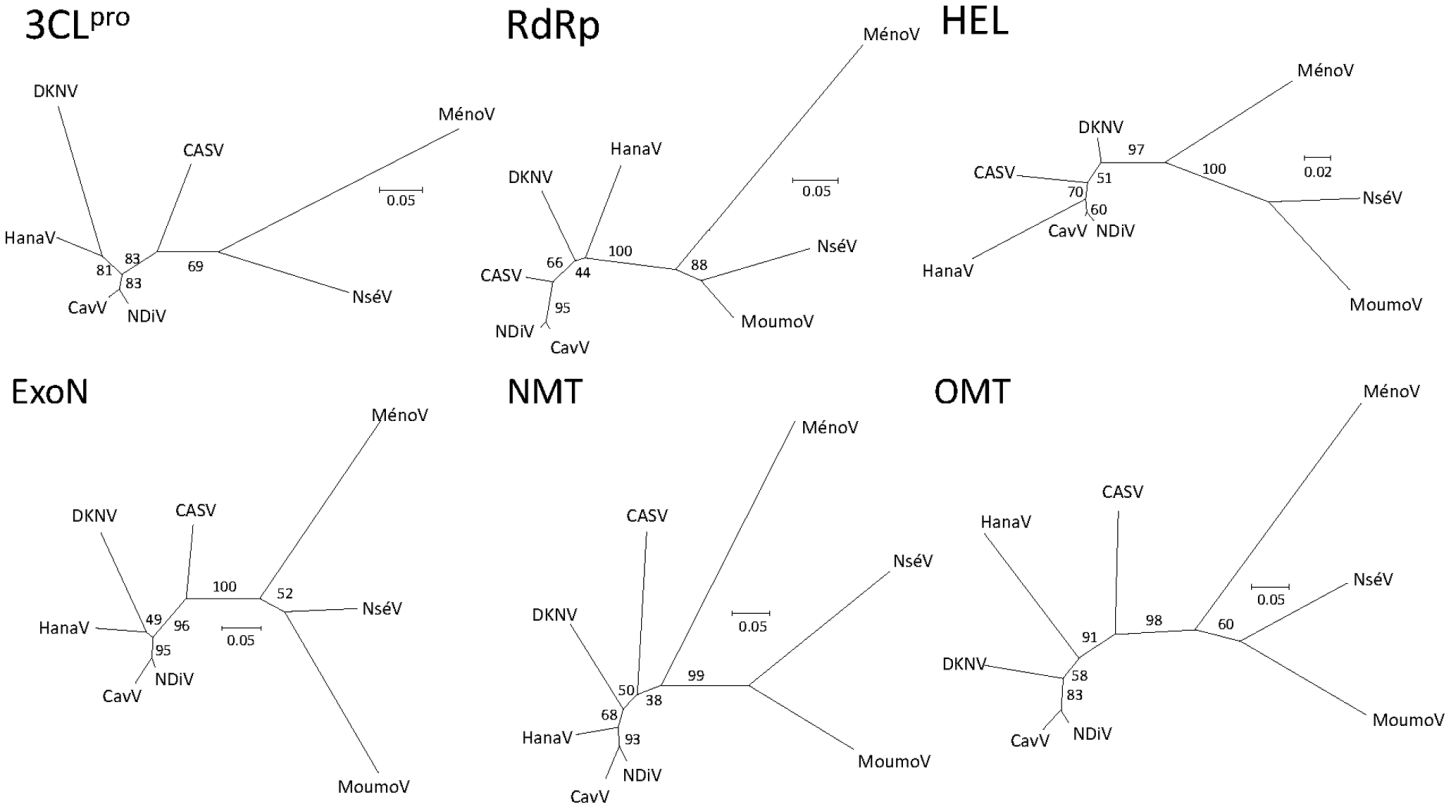
Phylogenetic analysis of the replicase domains of pp1ab. Unrooted phylogenies were generated from the protein sequences of the indicated replicase domains using the maximum likelihood method. Bootstrapping replicates (1000) are rounded to a value out of 100. The branch length is proportional to the evolutionary distance as shown by the scale bar (substitutions per amino acid site).

Unrooted phylogenetic trees were generated by a maximum likelihood using alignments of 3CL^pro^, RdRp, HEL, ExoN, NMT and OMT with the homologous domains of other mesoniviruses (Fig. 4). The generally high bootstrap values for the internal branches of trees generated from the replicase domains best supported a topology in which CASV was separated from its closest relative, alphamesonivirus 1, by the nodes of HanaV and DKNV. Similar results were obtained using a neighbor joining model (data not shown). Hence, as CASV does not cluster with alphamesonivirus 1, the tree topology further supports its inclusion as a new species of mesonivirus.

In this report we have performed sequence analysis of the first insect mesonivirus detected in Australia, designated CASV. Phylogenetic analysis of the CASV genome in combination with its isolation from a different genus of mosquitoes captured on a separate continent to previously described mesoniviruses, supports the classification of CASV as a new species in the *Mesoniviridae* family. The detection of mesoniviruses in the tropics of three different continents suggests that the viruses are geographically widespread. In agreement with this, we have also recently isolated new viruses with high sequence identity to NDiV from *Culex annulirostris* mosquitoes captured in Brisbane on the east coast of Australia (McLean, Hobson-Peters, Hall, van den Hurk et al., unpublished data). The relatively recent identification of this group of viruses is likely due to the lack of any disease association which meant there was previously little incentive to isolate these agents. Sequencing of more isolates will provide further insights into the evolutionary development of these recently classified viruses in addition to greater understanding of the mosquito virome.

## Supporting Information

Figure S1 Analysis of CASV virion diameter. Images of 133 CASV virions were selected following cryo-electron microscopy or uranyl acetate staining. The diameter of each virion was determined and graphed.
(TIFF)

Figure S2 Analysis for replication of CASV following inoculation onto various cell lines. Monolayers of various cell lines were inoculated with CASV or WNV at an M.O.I of 10. A) After 48 hr, the Vero and C6/36 cell monolayers were fixed in acetone and probed with a cocktail of two monoclonal antibodies specific for dsRNA (green). The nucleus of each cell was stained by Hoechst (blue). B) After five days, the cell monolayer was harvested and RNA extracted. RT-PCR using gene-specific primers was performed and products visualised following gel electrophoresis. M – marker; W – wash; V – Vero; B – BHK; S - SW13; D – DF-1; H – HSU; CB – Chao Ball; C6– C6/36.
(TIF)

Movie S1 Cryo-electron tomography tilt series. A movie through a tomogram of a representative virion with clearly visible surface projections. Scale bar 20 nm.
(AVI)
