# Supplementary figures and images for: A New Species of Mesonivirus from the Northern Territory, Australia
Source: PLoS One. 2014 Mar 26;9(3):e91103. doi: 10.1371/journal.pone.0091103 (PMC3966781; doi:10.1371/journal.pone.0091103)

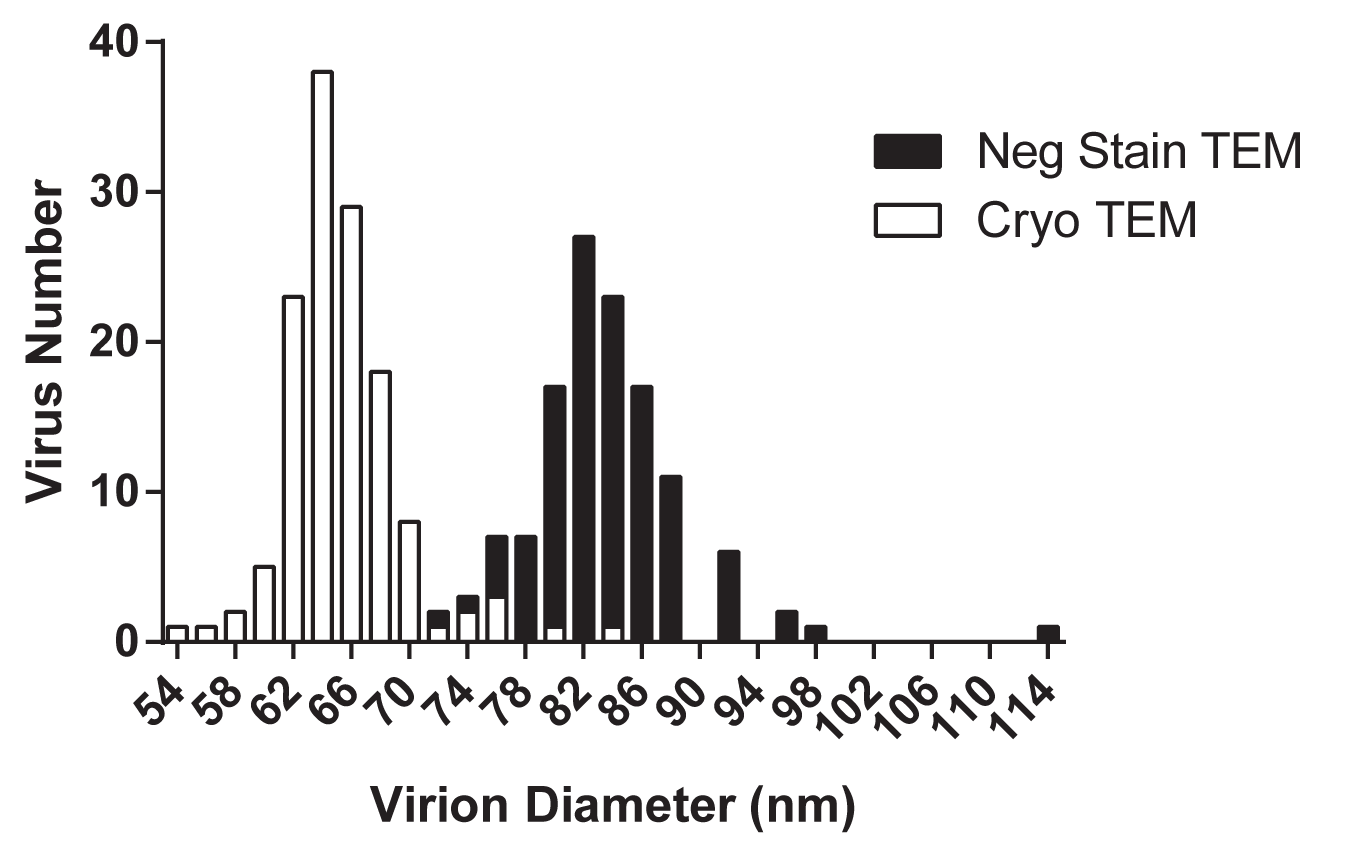

Supplement: Figure S1 — Analysis of CASV virion diameter. Images of 133 CASV virions were selected following cryo-electron microscopy or uranyl acetate staining. The diameter of each virion was determined and graphed. (TIFF) [file pone.0091103.s001.tiff]

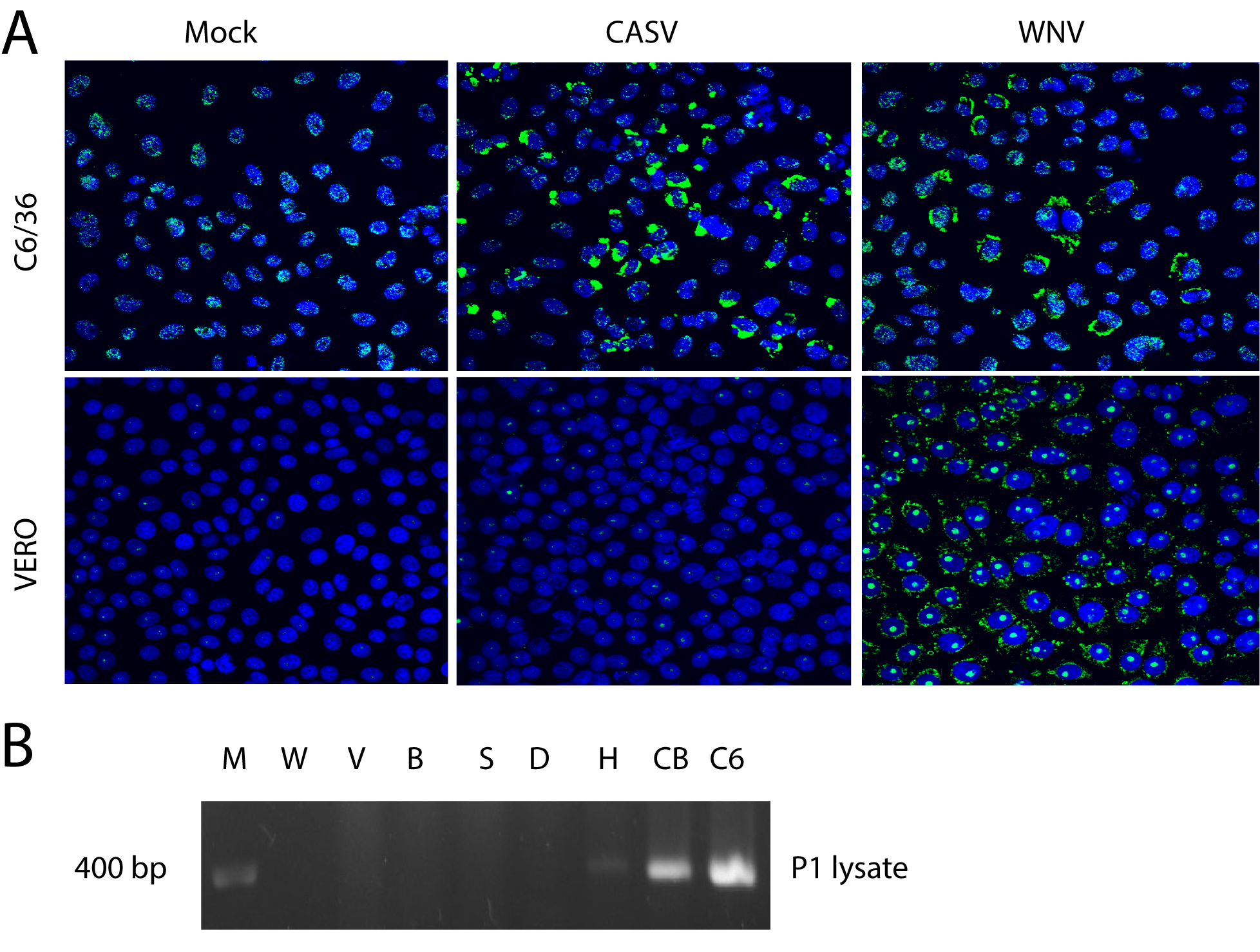

Supplement: Figure S2 — Analysis for replication of CASV following inoculation onto various cell lines. Monolayers of various cell lines were inoculated with CASV or WNV at an M.O.I of 10. A) After 48 hr, the Vero and C6/36 cell monolayers were fixed in acetone and probed with a cocktail of two monoclonal antibodies specific for dsRNA (green). The nucleus of each cell was stained by Hoechst (blue). B) After five days, the cell monolayer was harvested and RNA extracted. RT-PCR using gene-specific primers was performed and products visualised following gel electrophoresis. M – marker; W – wash; V – Vero; B – BHK; S - SW13; D – DF-1; H – HSU; CB – Chao Ball; C6– C6/36. (TIF) [file pone.0091103.s002.tif]
